# Skeletal age during hurricane impacts fluctuating asymmetry in Cayo Santiago rhesus macaques

**DOI:** 10.1002/ece3.10425

**Published:** 2023-08-11

**Authors:** Ashly N. Romero, Edwin Dickinson, Cassandra M. Turcotte, Claire E. Terhune

**Affiliations:** ^1^ Department of Anthropology University of Arkansas Fayetteville Arkansas USA; ^2^ Department of Basic Medical Sciences University of Arizona College of Medicine – Phoenix Phoenix Arizona USA; ^3^ College of Osteopathic Medicine New York Institute of Technology Old Westbury New York USA

**Keywords:** Cayo Santiago, developmental instability, fluctuating asymmetry, hurricane, *Macaca mulatta*, natural disaster, rhesus macaque, skeletal morphology

## Abstract

As natural disasters become more frequent due to climate change, understanding the biological impact of these ecological catastrophes on wild populations becomes increasingly pertinent. Fluctuating asymmetry (FA), or random deviations from bilateral symmetry, is reflective of developmental instability and has long been positively associated with increases in environmental stress. This study investigates craniofacial FA in a population of free‐ranging rhesus macaques (*Macaca mulatta*) that has experienced multiple Category 3 hurricanes since the colony's inception on Cayo Santiago, including 275 individuals from ages 9 months to 31 years (F = 154; M = 121). Using geometric morphometrics to quantify FA and a linear mixed‐effect model for analysis, we found that sex, age, and decade of birth did not influence the amount of FA in the individuals included in the study, but the developmental stage at which individuals experienced these catastrophic events greatly impacted the amount of FA exhibited (*p* = .001). Individuals that experienced these hurricanes during fetal life exhibited greater FA than any other post‐natal developmental period. These results indicate that natural disasters can be associated with developmental disruption that results in long‐term effects if occurring during the prenatal period, possibly due to increases in maternal stress‐related hormones.

## Graphical abstract

Fluctuating asymmetry (FA), or random deviations from symmetry, can be used as a proxy for developmental instability, and anthropogenic and natural disruptions have been associated with increased levels of FA. In this study, we investigate the effect of demographic variables (age, sex, and decade of birth) and hurricane experience on FA in a model primate taxon: rhesus macaques (*Macaca mulatta*) to better understand the impact major ecological catastrophes have on skeletal development. Individuals that experienced a hurricane event in utero exhibited higher levels of FA than juvenile or adult individuals that experienced that same event, which could be due to increased maternal stress hormones such as glucocorticoids that can disrupt fetal development.

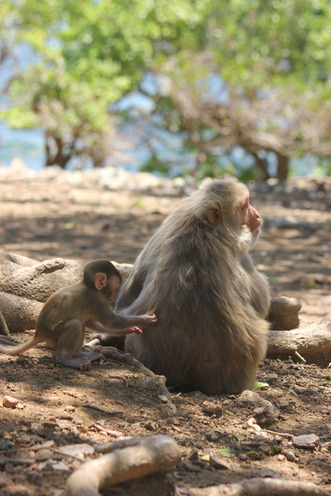

## Resumen

A medida que los desastres naturales se vuelven más frecuentes debido al cambio climático, entender el impacto biológico de estas catástrofes ecológicas en poblaciones silvestres va en aumento pertinente. La asimetría fluctuante (AF), o desviaciones aleatorias de simetría bilateral, es reflejo de inestabilidad durante el desarrollo y se ha asociado positivamente con incrementos en estrés ambiental durante mucho tiempo. Este estudio investiga AF craneofacial en una población de macacos rhesus (*Macaca mulatta*) en libertad que ha experimentado múltiples huracanes categoría 3 desde el inicio de la colonia en Cayo Santiago, e incluye 275 individuos de 9 meses a 31 años de edad (F = 154; M = 121). Usando morfometría geométrica para cuantificar AF y un modelo lineal de efectos mixtos para análisis, encontramos que el sexo, la edad y la década de nacimiento no influyeron en la cantidad de AF en los individuos incluidos en el estudio, pero la etapa de desarrollo en la que los individuos experimentaron estos eventos catastróficos impactó altamente la cantidad de AF exhibida (*p* = .001). Los individuos que experimentaron estos huracanes durante el período fetal exhibieron mayor AF que cualquier otro período de desarrollo posnatal. Estos resultados indican que los desastres naturales pueden asociarse con trastornos del desarrollo que tienen efectos a largo plazo si ocurren durante el período prenatal, posiblemente debido al aumento de hormonas maternas relacionadas con el estrés.

## 1 INTRODUCTION

Fluctuating asymmetry (FA)—defined as random deviations from symmetry in traits that are otherwise bilaterally symmetrical—has been repeatedly demonstrated to reflect a morphological proxy for the frequency and/or magnitude of stress events experienced by an individual (Badyaev et al., 2000; Lens et al., 1999; Polak, 2003; Sherman et al., 2009; Weller & Ganzhorn, 2004). As bilateral traits share a common genome (Klingenberg, 2015; Polak, 2003), the presence of FA is a manifestation of developmental instabilities that disrupt typical developmental patterns, resulting in the phenomenon of asymmetry (Møller, 1991; Palmer & Strobeck, 1986; Waddington, 1957). Though literature documenting FA is vast and variable, our understanding of how demographic factors such as age and sex influence FA—as well as when individuals may be most susceptible to developmental disruptions—remains ambiguous, particularly within the context of broad‐scale ecological catastrophes. This study investigates FA in a cross‐sectional, ontogenetic sample of free‐ranging rhesus macaques (*Macaca mulatta*) on the island of Cayo Santiago. The macaques in this sample span multiple generations and include individuals that experienced one or more Category 3 hurricanes during their lifetime on Cayo Santiago. We examine how FA changes across ontogeny, with demographic factors such as age and decade of birth and examine the impact of experiencing such a major natural disaster on FA levels.

Both anthropogenic and natural disruptions have been shown to impose stress on individuals, leading to the use of FA as an indicator of environmental stress levels (Clarke & McKenzie, 1992; Manning & Chamberlain, 1994; Söderman et al., 2007). For example, levels of FA in the mandibles of immature common shrews (*Sorex cinereus*) are significantly greater in populations subjected to environmental disturbance via industrial logging activity (Badyaev et al., 2000). Higher levels of FA were further associated with decreases in general fitness, measured via each individual's body mass (Badyaev et al., 2000). Similarly, habitat disturbance has been inferred to drive temporal increases in FA between historical and modern populations of endangered bird species, with levels of asymmetry reaching a sevenfold increase in highly degraded (i.e., deforested) localities (Lens et al., 1999). Furthermore, young mice in deforested environments with higher food scarcity exhibit higher levels of FA than adults (Díaz & Morán‐López, 2023). In addition to anthropogenic destruction, environmental change following a 1999 hurricane in Ohio (USA) was shown to increase levels of FA within populations of forest‐dwelling deer mice (*Peromyscus maniculatus*; Hopton et al., 2009). Indeed, hurricane and tornado events are well‐documented in driving changes in mortality profile, community structure, and fitness in both vertebrates (e.g., Gannon & Willig, 1994; Weidenfeld & Weidenfeld, 1995; Woolbright, 1996) and invertebrates (e.g., Willig & Camilo, 1991) by increasing food scarcity, altering local microclimates, or destratifying habitats through the loss of shrubbery and canopy coverage (Bellingham et al., 1995; Ney‐Nifle & Mangel, 2000; Wunderle Jr, 1995).

### 1.1 Other potential contributors to fluctuating asymmetry

Extrinsic disturbances are not the only mechanisms by which asymmetry is accumulated within the skeleton. As described by Hallgrímsson (1999), magnitudes of FA increase over ontogeny in both humans and nonhuman primates; this phenomenon was ascribed to the additive accumulation of asymmetrical mechanical factors (e.g., stresses placed on bones during locomotion or mastication) and undirected bone remodeling (e.g., drift) throughout an individual's life. While the first of these processes may arguably reflect directional asymmetry (as opposed to fluctuating asymmetry), morphological drift via a linearly increasing quantity of random deviations over time would predict an increase in FA within older individuals (Hallgrímsson, 1999). Bone remodeling is a maintenance process, involving the coordinated action of osteoclasts and osteoblasts to iteratively remove and replace skeletal tissue over time. As osteoblastic and osteoclastic activity are occurring at the same site, the potential for tangible morphological changes to be manifested is minimal. However, during bone modeling, opportunity for morphological changes increases due to bone deposition occurring independent of, or spatially separated from, bone resorption. As most bone modeling occurs prior to skeletal maturity, it is reasonable to infer that opportunities for FA to manifest via this mechanism are increased within developing individuals. To this end, the impact of developmental instabilities on FA is thought to be magnified during ontogeny, such that the impact of early‐life adversity may contribute more strongly to FA than hardship experienced when the skeleton has already been formed (Gluckman & Hanson, 2006; Hallgrímsson, 1999). This theory is substantiated by recent work into the human cranium, which highlights a window of vulnerability to developmental instability occurring between 1 and 5.5 years of age, with a uniquely sensitive time between 4 and 5.5 years (Moes et al., 2022).

In addition to age, other demographic variables—most notably sex—have been hypothesized to impact skeletal FA, with varying degrees of support. Measurements of cranial FA in humans and nonhuman primates have largely yielded no sex‐specific patterns (e.g., Hallgrímsson, 1993, 1999; Van Dongen, 2015), with two notable exceptions: an increase in osseous nasal FA in male humans from multiple populations as compared to females (Schlager & Rüdell, 2015) and a similar increase in overall cranial FA in male gorillas relative to females (Romero et al., 2022). Beyond primates, sex was not observed to drive differences in FA within either hurricane‐affected or control‐group deer mice (Hopton et al., 2009), nor in red squirrels occupying either disturbed or undisturbed woodland habitats (Wauters et al., 1996). Sex is similarly reported as a non‐significant factor upon FA within South American water rats (Caccavo et al., 2021), long‐tailed spiny rats, hairy‐tailed akodonts, wooly mouse opossums, or Amazonian red‐sided opossums (Castilheiro et al., 2022). Finally, among Italian wall lizards, sex‐based differences in FA are observed in femoral pore distribution, but not in head shape (Simbula et al., 2021), and sex‐based differences in FA are found in the mandible of common shrews exposed to habitat disturbance (Badyaev et al., 2000).

### 1.2 Study aims

In this study, we use a free‐ranging sample of rhesus macaques (*Macaca mulatta*) from Cayo Santiago, Puerto Rico, to assess three distinct aims: (1) clarify the relationship between FA and age within a model primate taxon; (2) quantify the potential role of other demographic variables—specifically sex and decade of birth—in driving FA; and (3) assess the impact of a catastrophic natural event (namely the landfall of two devastating hurricanes in 1989 and 1998, respectively) upon FA levels in a free‐ranging primate population.

## 2 MATERIALS AND METHODS

### 2.1 Sample composition

**FIGURE 1:**
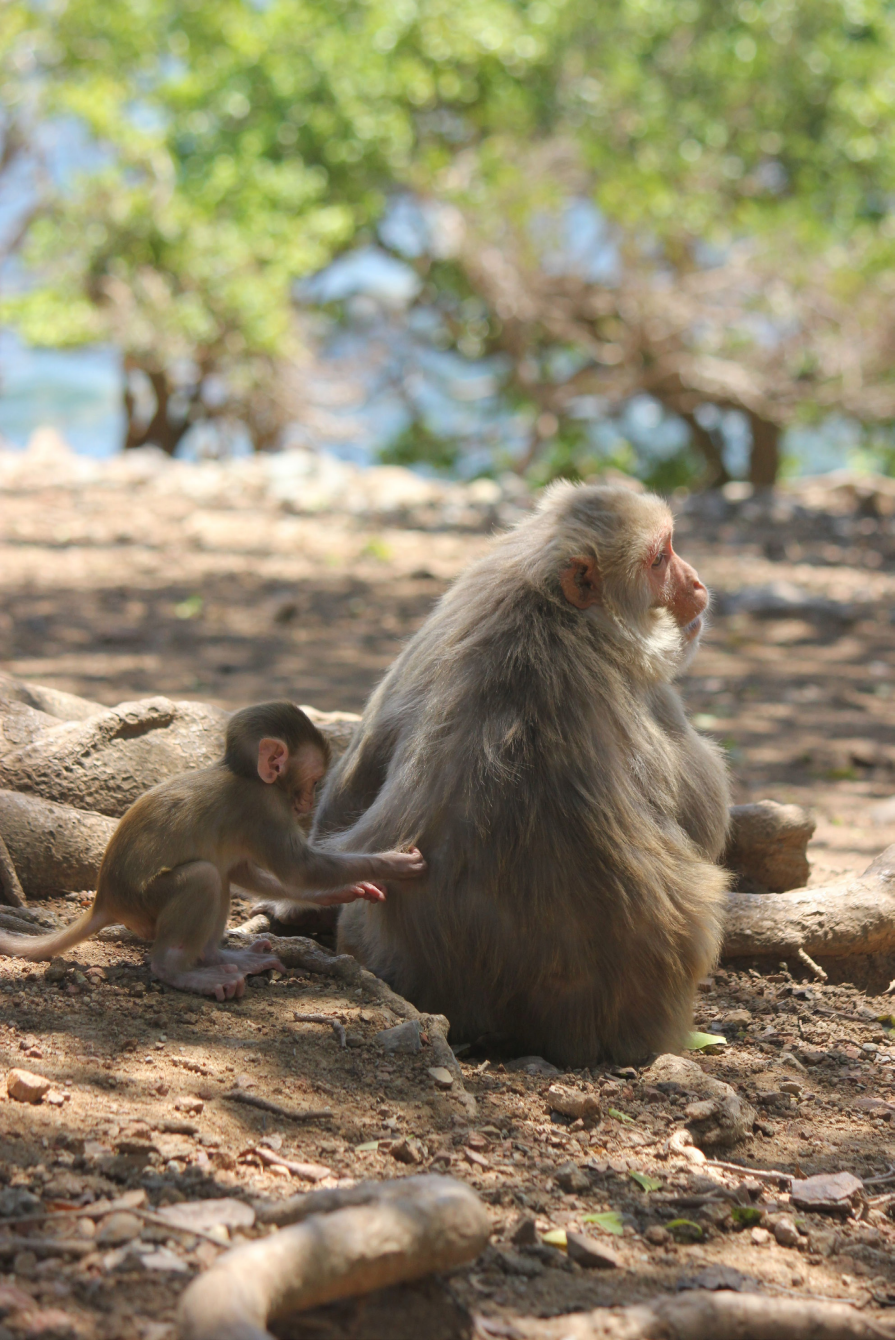
Adult and juvenile rhesus macaques on the island of Cayo Santiago.

Our sample derives from the free‐ranging rhesus macaque colony of Cayo Santiago (Figure 1), where a group of 409 rhesus macaques were originally transported to the island in 1938 from point of capture in India (Carpenter, 1971). Over the past century, the population grew to its current level of 1800 individuals. After death, the bodies of all animals are collected, macerated, and stored long‐term at the University of Puerto Rico Recinto de Ciencias Médicas.

Cayo Santiago is an 18.2‐hectare island off the coast of Puerto Rico, characterized by a tropical environment with no predators. From 1950 to 2012, the island has experienced two named hurricanes: Hurricane Hugo in 1989 and Hurricane Georges in 1998. Both Hugo and Georges were Category 3 hurricanes at landfall. In each case, the island experienced little loss of primate life but suffered significant ecological damage in the form of vegetation and infrastructural loss.

The Caribbean Primate Research Center (CPRC) oversees the health and maintenance of the colony, which is otherwise free ranging. Once commercial primate diets were produced in the United States, the CPRC began provisioning the macaques with fresh water and monkey chow and have increased supplementation plans in recent years due to hurricane‐related environmental instability (Kessler & Rawlins, 2016).

We analyzed crania from 275 individuals of both sexes (female = 154; male = 121; Table 1). This sample is cross‐sectional, containing individuals aged from 9 months to 31 years, and represents animals born across six decades (1951–2005). The sample is further subset into animals that did not experience a hurricane (*n* = 174; F = 90, M = 84) and animals that experienced at least one named hurricane in their lifetime (*n* = 101; F = 64, M = 37). Of the latter group, 78 animals experienced just one hurricane and 23 animals experienced two. To better understand the effect of adversity on the ontogeny of fluctuating asymmetry, the individuals who experienced a hurricane were further divided into groups on the basis of skeletal age at which the hurricane was experienced: fetal (*n* = 10) individuals who experienced the hurricane prenatally; juvenile (*n* = 50) individuals who experienced the hurricane prior to skeletal maturity (<8 year in males, <15 year in females); adult (*n* = 41) individuals who were skeletally mature during the hurricane event (>8 year in males, >15 year in females).

**TABLE 1:** Sample composition.

Sex	
Female	157
Male	121
Hurricane experience	
Yes	101
No	174
Skeletal age at hurricane	
Fetal	10
Juvenile	50
Adult	41

*Note*: The number of individuals that are male and female, experienced a hurricane or did not, and the number of individuals that experienced a hurricane at the fetal, juvenile, or adult stage of skeletal development.

### 2.2 Data collection and processing

**FIGURE 2:**
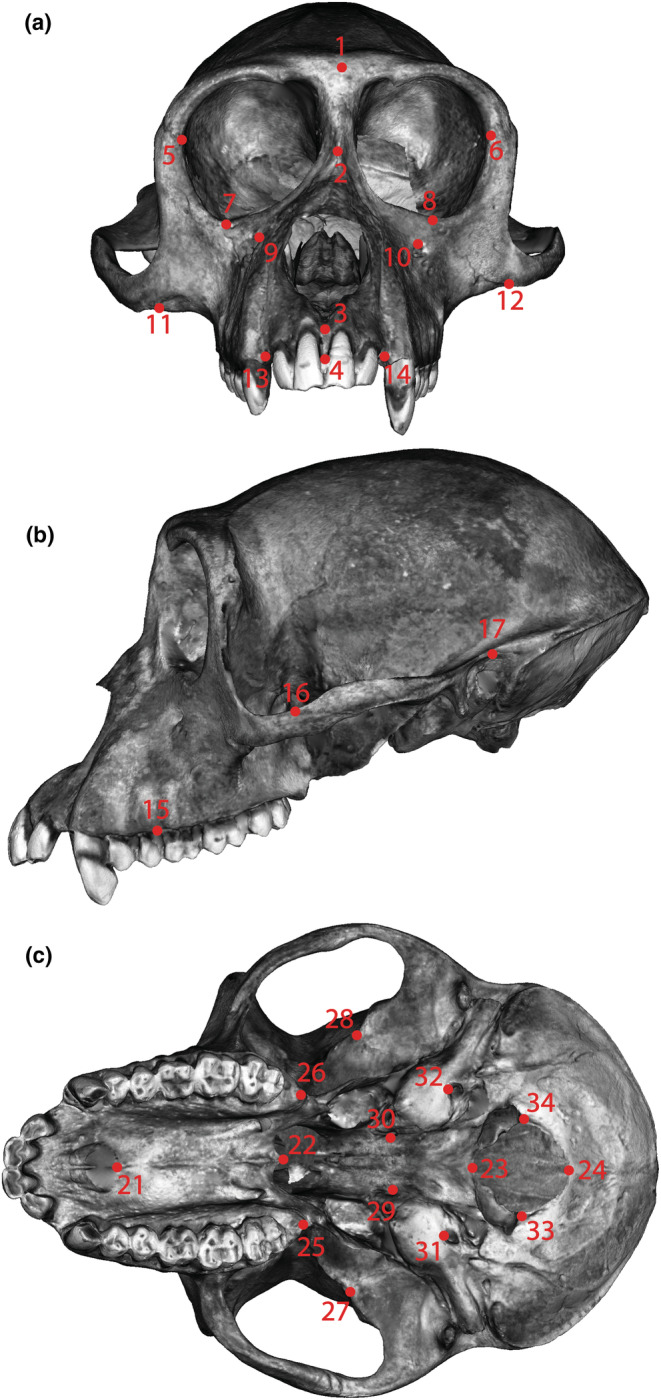
Landmarks used in this study on the (a) anterior view, (b) left lateral view, and (c) inferior view of a female rhesus macaque (CPRCMUS‐04439). Landmark definitions can be found in Table 2.

Crania were 3D‐scanned in Puerto Rico using an HDI 120 blue LED scanner (LMI Technologies). After scanning, the 3D surface models were processed in Geomagic Studio (3D Systems) using the “fill holes” and “mesh doctor” functions. After processing, the 3D models were imported into 3D Slicer (Version 4.11.20210226; Fedorov et al., 2012) for landmarking. For better visualization of anatomically based landmarks on the 3D models (e.g., sutural intersections, foramina), the “display” settings in the “models” module were adjusted to make the “scalars” visible, the “active scalar” RGB, and the “scalar range mode” direct color mapping. This overlays the 3D model with surface images collected during the scanning process. A total of 34 fixed landmarks (13 bilateral landmark pairs, plus 8 midline points) were placed on the cranium using the “fiducial markups” function in the “markups” module of 3D Slicer (Table 2; Figure 2). These landmark configurations were then exported as .fcsv files, imported into R (R Core Team, 2020) and collated, and then saved as .tps files for analysis in MorphoJ (Klingenberg, 2011). Landmarks were placed twice on each of the 275 individuals in the sample to include an error effect during data analysis.

**TABLE 2:** Landmarks.

Landmark	Midline/bilateral	Location	Description
1	Midline	Face	Nasion (point where two nasal bones and frontal bone intersect)
2	Midline	Face	Premaxillary midline suture (superior point)
3	Midline	Face	Nasospinale (midpoint on lower border of nasal aperture)
4	Midline	Face	Alveolare (inferior tip of bone between upper central incisors)
5, 6	Bilateral	Face	Frontozygomatic suture at orbital rim
7, 8	Bilateral	Face	Zygomaxillare superior
9, 10	Bilateral	Face	Infraorbital foramen (most medial and superior point)
11, 12	Bilateral	Face	Zygomaxillare inferior
13, 14	Bilateral	Face	Premaxilla‐maxilla junction at alveolus
15, 18	Bilateral	Face	Midpoint on alveolus between the 4th premolar and the first molar
16, 19	Bilateral	Face	Temporozygomatic suture (superior point)
17, 20	Bilateral	Face	External auditory meatus (most superior point)
21	Midline	Face	Incisive fossa (most posterior and inferior point on the incisive fossa; between incisive foramina when there are two)
22	Midline	Face	Interpalatine suture (posterior point)
23	Midline	Base	Basion (anterior margin of foramen magnum)
24	Midline	Base	Opisthion (posterior margin of foramen magnum)
25, 26	Bilateral	Face	Maxillary tuberosity (intersection of maxilla and palatine)
27, 28	Bilateral	Face	Sphenosquamosal suture along infratemporal crest
29, 30	Bilateral	Base	Lateral joining of spheno‐occipital suture
31, 32	Bilateral	Base	Carotid canal (anterior point)
33, 34	Bilateral	Base	Posteromedial junction of occipital condyle and foramen magnum

*Note*: Description of the 34 landmarks used in this study.

### 2.3 Quantification of fluctuating asymmetry

A Procrustes superimposition or Procrustes fit was performed in MorphoJ, which takes the landmark configurations and reflects the bilateral landmarks across the midline. The new, mirrored landmark configurations are relabeled, and then both the original and mirrored landmark configurations are translated, rotated, and scaled to the same position, orientation, and size using a least squares approach (Dryden & Mardia, 1998; Goodall, 1991; Gower, 1975; Klingenberg, 2015). Then, the symmetric and asymmetric component of shape are estimated (Kent & Mardia, 2001; Klingenberg et al., 2002; Mardia et al., 2000). Estimating the asymmetric component calculates the equivalent of the distance between the original and mirrored landmark pairs using the sum of squared distances.

A Procrustes ANOVA (analysis of variance) was then performed in MorphoJ to determine the levels of FA present in each individual's cranium. This analysis includes individuals (specimens) and sides (right/left) as main effects, as well as an interaction term between individual and side (individual*side). The average difference between the right and left sides represents directional asymmetry and the individual‐by‐side term represents FA (Klingenberg et al., 2002; Klingenberg & McIntyre, 1998; Palmer & Strobeck, 1986). This model also includes the replicate landmark configurations to quantify measurement error and assess error in relation to FA signal. Ideally, measurement error should be low for studies of FA to optimize the signal‐to‐noise ratio. Measurement error is calculated in this model from the variation among the replicate landmark configurations (Klingenberg et al., 2002). Dividing the Procrustes mean squares of the individual‐by‐side term (FA) by the Procrustes mean squares of the measurement error term results in the *F*‐value, or *F* ratio, with which one can assess FA signal and noise from measurement error (Klingenberg, 2015). Terms were considered statistically significant at α = 0.05 or below. The mean squares in the Procrustes ANOVA were used to calculate the percent of variation that each term in the model contributed to overall variation in the sample (Gómez‐Robles et al., 2013). The output of the Procrustes ANOVA from MorphoJ includes Procrustes FA scores that were used for further analysis. Procrustes FA scores rather than Mahalanobis FA scores were used because the latter metric requires large sample sizes to reliably estimate the covariance matrix and are difficult to interpret due to their lack of comparability to other measures of shape variation (Klingenberg, 2015; Klingenberg & Monteiro, 2005). After extracting the Procrustes FA scores for each individual in the dataset, all further analyses were performed in R.

### 2.4 Statistical analysis

To assess drivers of FA, several iterations of a linear mixed‐effect model were constructed using *R* (R Core Team, 2020) with the packages “lmerTest” (Kuznetsova et al., 2017) and “lme4” (Bates et al., 2015). To first assess the potential relationship of age and demography to FA (Aims 1 and 2), we constructed a model containing age, sex, and decade of birth as fixed effects, while accounting for the potential confounding influence of matriline as a random effect, following Winter (2013) and Bates et al. (2015). This model was run on two subsets of the FA scores: first the full dataset including all individuals (*n* = 275) and then a second subset of 174 individuals that had never experienced a hurricane to mitigate any potential influence of environmentally driven FA upon these results. Post hoc Tukey tests utilizing Bonferroni‐Holm correction were subsequently applied using the *R* package “comptest” (Hothorn et al., 2016). Terms were considered statistically significant at α = 0.05 or below for these and all further analyses.

To assess Aim 3, we constructed three separate linear mixed‐effect models using the previously mentioned packages to investigate how experiencing a hurricane may alter FA. The first model was run on the entire dataset (*n* = 275) and included age (continuous, using age at death data provided by the CPRC), sex, decade of birth, and hurricane yes/no (a Boolean summary of whether an individual had, or had not, experienced a hurricane in its lifetime; Yes = 101; No = 174) as fixed effects, and matriline as a random effect. The second model sought to investigate whether experiencing multiple hurricanes had an additive effect, and included age, sex, decade of birth, and number of hurricanes experienced in an animal's lifetime (0: *n* = 174; 1: *n* = 78; 2: *n* = 23) as fixed effects, and matriline as a random effect. Finally, we explored whether experiencing a hurricane at different periods of ontogeny influenced the development of FA. This model was run only on animals that had experienced a hurricane (*n* = 101) and included age, sex, decade of birth, and age at hurricane (fetal, juvenile, adult) as fixed effects, and matriline as a random effect. Thus, all models contained 4 fixed effects (age, sex, decade of birth, and 1 hurricane‐related variable) and 1 random effect (always matriline). A simulated power analysis (see Diggle et al., 2002; Green & MacLeod, 2016) given our sample size and model complexity suggests the ability to assess effect sizes of ~5.1% with 80% power (used as a traditional threshold for analyses of this nature; see Field et al., 2007 for discussion).

## 3 RESULTS

**FIGURE 3:**
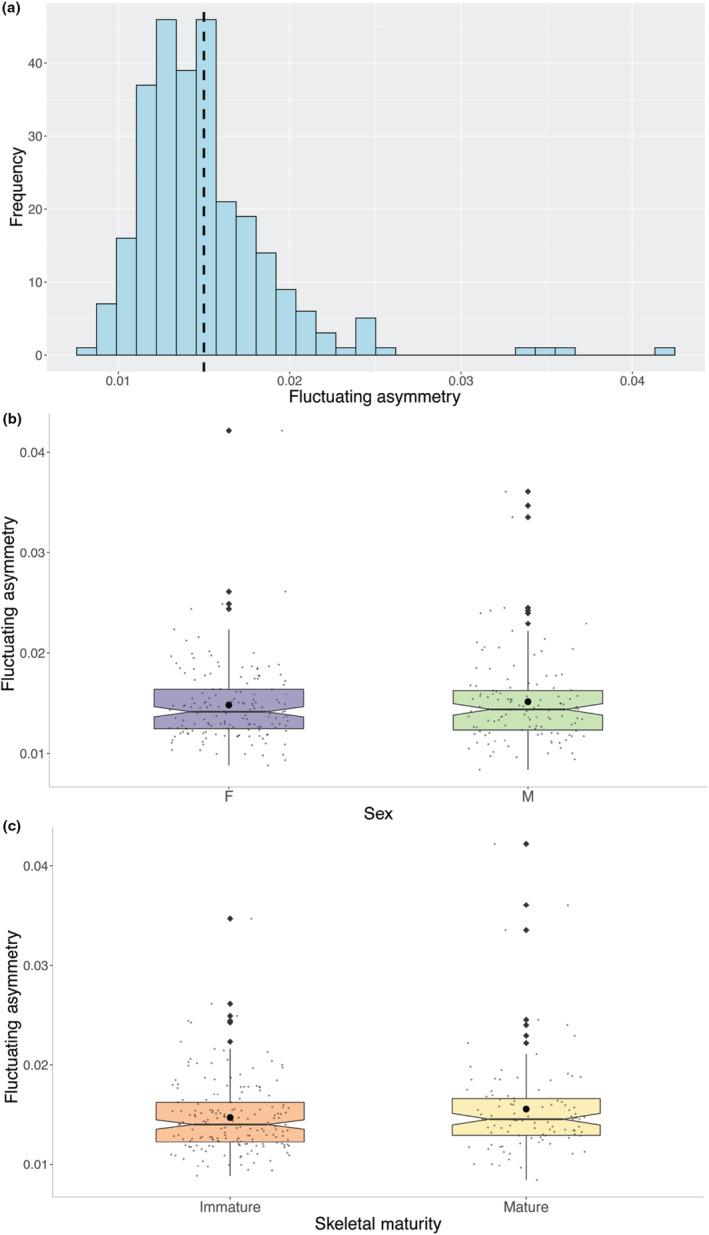
Plots illustrating the distribution of raw data in a (a) histogram showing the frequency and mean (dashed black line) of FA values, (b) boxplot of FA values separated by sex showing the mean (solid round point) value of males and females, and (c) boxplot of FA values separated by skeletal maturity showing the mean (solid round point) value of skeletally mature and immature individuals.

Both directional asymmetry (DA) and FA are present in the sample (*p* < .001 for all; Table 3). Most shape variation in the sample comes from variation between individuals (91.06%; Table 3). This high level of individual variation can be attributed to variation between the left and right averages of landmark positions for the individuals in the sample (Klingenberg, 2015). Measurement error accounts for 5.04% of the variation in the sample, which is slightly higher than the variation attributed to FA. However, this error is calculated in the Procrustes ANOVA model with DA and FA, and FA remains clearly statistically significant in the sample regardless (*p* < .001). The *F* ratio of FA to measurement error Procrustes mean squares is 1.54, which illustrates that the FA signal is 1.54 times greater than measurement error. This is enough to be confident that our results are valid with an *F* ratio comparable to other published studies (Hopton et al., 2009; Quinto‐Sánchez et al., 2015; Romero et al., 2017; Simbula et al., 2021). The Procrustes FA scores extracted from MorphoJ had a mean of 0.015, median of 0.014, and standard deviation of 0.004. Distribution of the data can be observed in Figure 3, where the frequency of FA scores is shown in a histogram (A) and the distribution of FA scores is illustrated by sex (B) and skeletal maturity at death (C). While mean FA is not comparable to other studies (because each Procrustes superimposition is unique), the variance and standard error here are slightly lower than those reported for *Macaca fascicularis* in Romero et al. (2022). This could be because the sample size in our study is much larger and thus provides a more accurate reflection of species‐level variation.

**TABLE 3:** Procrustes ANOVA table.

Effect	df	SS	MS	*F*	*p*	% var
Individual	13,974	3.38149449	0.0002419847	21.91	<.001*	91.06%
Side (DA)	44	0.01183859	0.0002690589	24.36	<.001*	0.32%
Individual*Side (FA)	12,056	0.13313906	0.0000110434	1.54	<.001*	3.59%
Error	26,125	0.18699523	0.0000071577			5.04%

*Note*: Results of the Procrustes ANOVA performed on all landmark configurations after a Procrustes fit. The side effect represents the directions asymmetry (DA) in the sample, and the individual*side effect represents fluctuating asymmetry (FA). The percent variation that each effect contributes to the sample is calculated in the last column (% var). Asterisk notes statistically significant relationships at the α = 0.05 level.

### 3.1 Fluctuating asymmetry as a product of age or demography

No significant effect of age at death on FA is observed in either our full dataset (*t =* 1.077, *p* = .282, df = 275; Table 4) or subset of macaques that did not experience a hurricane (*t* = −1.26, *p* = .209, df = 174). Sex (*t* = 0.896, *p* = .371, df = 274.8 for full sample; *t* = 0.853, *p* = .395, df = 174 in subset model) and decade of birth (*t* = 0.960, *p* = .339, df = 132.1 in full sample; *t* = 0.221, *p* = .825, df = 174 in subset model) are similarly non‐significant throughout.

**TABLE 4:** Statistical parameters derived from linear mixed‐effect models demonstrating the importance of various fixed effects (age, sex, decade of birth, hurricane Yes/No, # of hurricanes, and age at hurricane) on FA score while controlling for matriline as a random effect.

Model	Response	Fixed effect	Estimate (Procrustes distance units)	Standard error	df	*t*‐value	*p*‐value
Model 1, All individuals (FA ~ Age + Sex + Decade of Birth + Hurricane Y/N + 1|Matriline)	FA Score	Age	0.00002	0.00172	275.0	0.428	.669
		Sex	0.00045	0.00005	274.8	0.877	.381
		Decade of Birth	0.00001	0.00002	245.5	0.347	.729
		Hurricane (Y/N)	0.00058	0.00071	255.4	0.823	.411
Model 2, No Hurricane Sample (FA ~ Age + Sex + Decade of Birth + 1|Matriline)	FA Score	Age	0.00008	0.00006	174.0	−1.260	.209
		Sex	0.00045	0.00053	174.0	0.853	.395
		Decade of Birth	<0.00001	0.00002	174.0	0.221	.825
Model 3, All individuals (FA ~ Age + Sex + Decade of Birth + # of Hurricanes + 1|Matriline)	FA Score	Age	0.00003	0.00006	275.0	0.481	.631
		Sex	0.00044	0.00051	274.9	0.850	.396
		Decade of Birth	0.00001	0.00002	242.4	0.371	.711
		# of Hurricanes (1)	0.00060	0.00071	0.0	0.843	.400
		# of Hurricanes (2)	0.00036	0.00128	0.0	0.299	.765
Model 4, Hurricane Experienced Sample (FA ~ Age + Sex + Decade of Birth + Age at Hurricane + 1|Matriline)	FA Score	Age	0.00019	0.00011	99.4	1.691	.094
		Sex	0.00198	0.00124	97.4	1.591	.115
		Decade of Birth	−0.00006	0.00007	95.9	−0.811	.419
		Age at Hurricane (1)	−6.e00e‐3	0.00172	100.5	−3.671	<.001*
		Age at Hurricane (2)	−0.00703	0.00215	99.0	−3.271	.001*

*Note*: Reference variable for sex = Female; reference variable for decade of birth = 1950s; reference variable for # of hurricanes = 0; reference variable for age at hurricane = fetal. Asterisk notes statistically significant relationships at the α = 0.05 level.

### 3.2 The impact of natural disasters on the development of fluctuating asymmetry

Modeling hurricane experience as a binary effect, where an animal either did or did not experience an event, has no significant effect on FA (*t* = 0.823, *p* = .411, df = 255.8; Table 4). Furthermore, no differences are found between individuals that had experienced 0 vesus 1 versus 2 hurricanes (*t* = 0.343, *p* = .710, df = 268.5).

However, among individuals that had experienced hurricanes, age at the time of the hurricane yields a significant effect on FA (*t* = 6.986, *p* = .001, df = 99.2). A post hoc, Bonferroni‐Holm adjusted Tukey's test demonstrated that fetal individuals during a hurricane event exhibit significantly greater FA later in life than those that were either juveniles (z = 3.687, *p* < .001, est. 0.006 ± 0.001) or adults (z = 3.313, *p* = .002, est. 0.007 ± 0.002) during the hurricane; however, no differences are observed between individuals that experienced a hurricane as juveniles versus adults (z = 0.622, *p* = .534, est. <0.001 ± 0.001). To further confirm this observation, we then ran a final model post hoc comparing levels of FA in individuals that had experienced a hurricane at a fetal age to all individuals that had not experienced a hurricane. FA levels were again significantly higher within individuals that had experienced a hurricane at a fetal age (*t* = 3.672, *p* < .001, df = 274.75) while no other fixed effects (age, sex, and decade of birth) attained significance.

## 4 DISCUSSION

**FIGURE 4:**
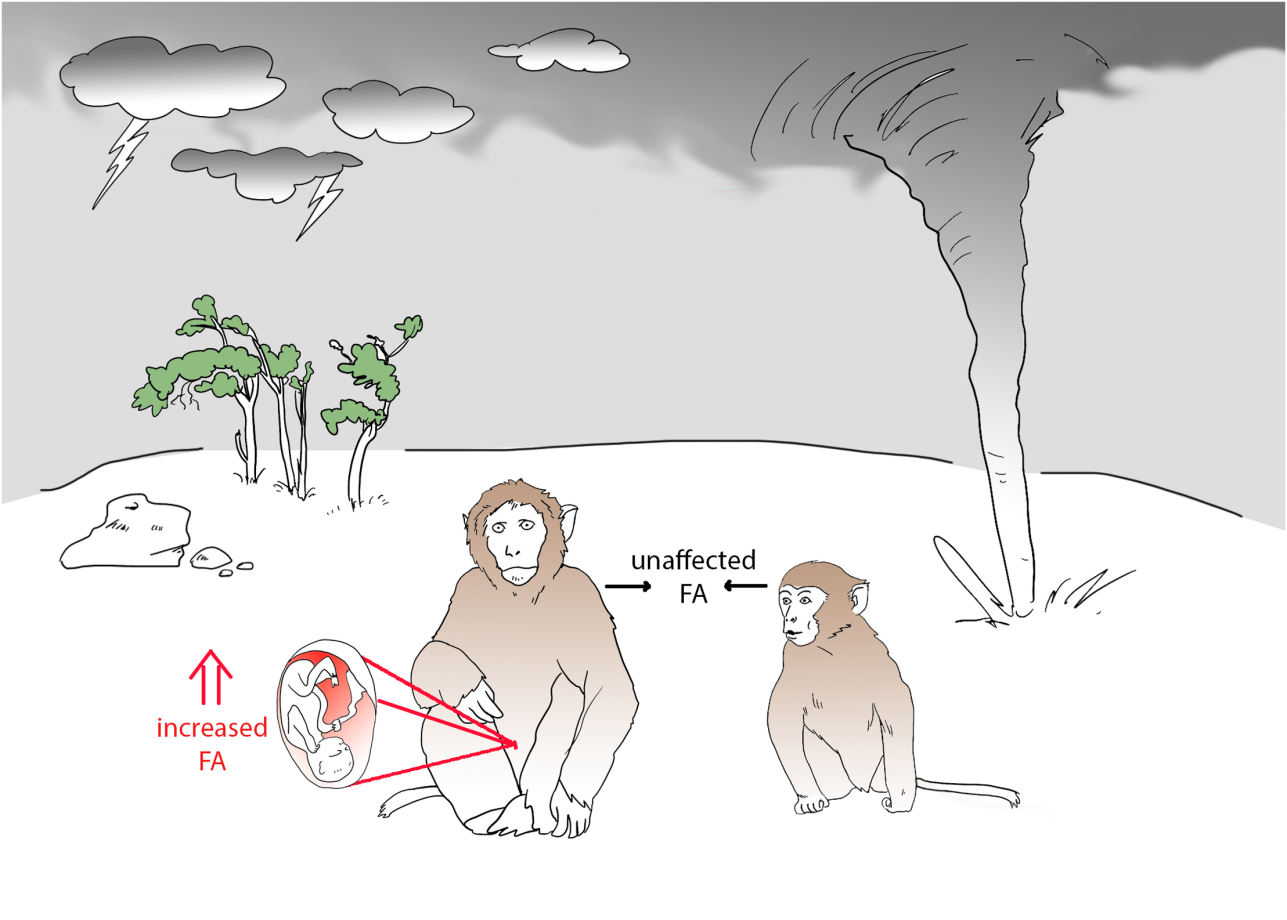
Graphic illustration of the results of this study showing that FA is increased in rhesus macaques that experienced a hurricane in utero.

In an assessment of the influence of age (Aim 1), sex and decade of birth (Aim 2), and natural disaster experience on FA (Aim 3), our results indicate that age, sex, and decade of birth have no statistical influence on FA in the population of rhesus macaques living on Cayo Santiago. While a binary hurricane experience factor did not appear to influence levels of FA, the developmental period in which an individual experienced a hurricane had a significant impact on FA levels later in life. Specifically, individuals that experienced hurricanes during fetal development exhibit significantly higher levels of FA than those that experienced a hurricane during either the juvenile or adult postnatal periods (Figure 4).

### 4.1 Sex and fluctuating asymmetry

These results support earlier findings that sex has little influence on FA in the Cayo Santiago macaque population (Hallgrímsson, 1999), which aligns with many studies on FA across animal clades (Caccavo et al., 2021; Castilheiro et al., 2022; Hallgrímsson, 1993; Hopton et al., 2009; Landi et al., 2021; Van Dongen, 2015; Wauters et al., 1996) but does not align with a handful of studies in humans (Schlager & Rüdell, 2015), gorillas (Romero et al., 2022), olive baboons (Romero et al., unpublished data), lizards (Simbula et al., 2021), and shrews (Badyaev et al., 2000). These studies used a variety of data collection methods (e.g., caliper measurements, 2D photographs, 3D landmark patches) and measured different body components (e.g., mandibles, crania, femoral pores), making consistency impossible and comparisons relatively difficult. It is possible that FA is more prevalent in particular traits, causing the range of results on sex‐specific FA. For example, traits that exhibit high levels of sexual dimorphism may also exhibit greater FA. Furthermore, the present study accounts for many factors that are not known in most populations (exact age at death, decade of birth, natural disaster experience, matriline, social group, etc.). Any subtle signal for sex‐specific FA may be overwhelmed by the inclusion of these other, more strongly correlated factors.

### 4.2 Aging and fluctuating asymmetry

Unlike the previous study of FA in this population (Hallgrímsson, 1999), our results indicate that there are no age‐associated increases in FA in the Cayo Santiago rhesus macaques. This phenomenon was ascribed by Hallgrímsson (1999) to multiple potential factors, including the cumulative effects of asymmetrical mechanical factors such as a side preference in chewing, and a tendency for bone form to drift through undirected remodeling, though chewing side preference has since been shown to be a minor contributor to FA levels (McGrath et al., 2022). Disagreements between this study and our own are potentially attributable to differences in methods or sample composition. To address this first point, it is important to note that the data presented by Hallgrímsson (1999) used linear measurements to quantify FA, as this study predated readily accessible 3D technology for geometric morphometric analyses. Linear measurements include less information than 3D landmarks in terms of position and, therefore, are potentially less accurate in quantifying FA. In terms of sample, meanwhile, this previous study did not include individuals that had experienced both hurricane Hugo and Georges, and further did not account for any potential impact of hurricane experience during analysis. Finally, from an analytical perspective, the original study did not attempt to account for inter‐relatedness of individuals by controlling for matriline, a method used within this study. In all, while an important first step in quantifying FA in the macaque and human skeleton, the present study includes additional information unavailable to Hallgrímsson (1999) and updated techniques that we feel render it a more accurate representation of FA in macaques than the previous work.

Age‐related increases in fluctuating asymmetry are also variably supported outside of macaques. Within moose, FA of the antlers is reported to be lowest in young calves (1–2 years of age); however, no significant differences were observed between age classes older than 2 years (Solberg & Saether, 1993). This suggests that magnitudes of FA do increase after birth but may plateau relatively early in life. The authors also observe that, for a given antler size, larger bulls exhibited less FA than relatively smaller bulls, suggesting that the ability to buffer environmental stress is improved in larger body‐sized individuals, an oft‐cited measurement of individual fitness. Within developing humans, meanwhile, both cranial and postcranial FA reduce with age until ~10 years of age, then increase during adolescence to peak at 13–14 years, before subsequently reducing until 18 years of age (Wilson & Manning, 1996). Similarly, Hope et al. (2013) observed that manual asymmetry decreased between the ages of 4–8, plateaued during early adolescence and further decreased after 13 years of age. The disruption to a general trend of reducing FA with age that occurs during adolescence is attributed in both studies to hormonal changes and rapid growth coincident with the onset of puberty. Alternatively, however, both Kobyliansky and Livshits (1989) and Penke et al. (2009) report that extreme senescence (>80 years of age) was associated with elevated FA in human populations. Thus, it is possible that age‐related increases in FA may be associated only with the extremes of old age, as opposed to a linear accumulation of asymmetry throughout life. This hypothesis could be tested in more diverse populations of nonhuman primates to further explore the nature of any potential relationship.

### 4.3 Prenatal vulnerability to natural disasters

Prenatal growth is characterized by the greatest velocity of bone growth, as the template for adult skeletal morphology is quickly laid down. Accordingly, perturbations—such as the stress experienced during and immediately following natural disasters—can have major consequences for the physical formation of bony structures (Liu et al., 2016). Notably, maternal stress—either nutritional or psychological—can be transmitted to the gestating fetus. For example, the hypothalamic–pituitary–adrenal (HPA) axis is a neuroendocrine mechanism through which the body regulates psychological stress, such as the experience of a hurricane (Smith & Vale, 2006). The end product of the HPA axis is production of cortisol, a hormonal biomarker commonly used as a proxy for stress (Bergman et al., 2010; Davis & Sandman, 2010; Rothenberger et al., 2011). Approximately 3% of maternal cortisol is transferred to fetal circulation (Stirrat et al., 2018) via the placenta (Argyraki et al., 2019), and excessive fetal exposure has been demonstrated to dysregulate the fetal HPA axis and disrupt tissue development (Argyraki et al., 2019; Provencal & Binder, 2015).

Additionally, high levels of maternal glucocorticoids can degrade the integrity of the placenta itself, disrupting placental transport of key histone modifiers and altering the landscape of fetal methyl bioavailability (Argyraki et al., 2019; Hogg et al., 2012; Myatt, 2006). In this way, maternal stress can have a life‐long impact on the skeleton of the offspring (Bateson, 2001; Gluckman et al., 2008; Morgan et al., 2005). Specifically, prenatal glucocorticoid overexposure alters histone acetylation and DNA methylation (Weaver et al., 2004). Methylation of the regulatory regions involved in the WNT/β‐catenin signaling pathway dysregulate osteoclast formation as well as the process of osteoblast differentiation (Bocheva & Boyadjieva, 2011). Meanwhile, disruption to the RANKL/RANK/OPG signaling pathway has been linked to deleterious changes in bone mineral density, which negatively impact fetal bone development and may predispose individuals to senescent disorders such as osteoporosis (Bocheva & Boyadjieva, 2011). Such mechanisms likely explain the role of catastrophe‐induced maternal stress in driving prenatal morphological disruptions such as those manifested as fluctuating asymmetry. Previous studies have shown that habitat destruction impacts FA levels in a variety of animals (e.g., Badyaev et al., 2000; Hopton et al., 2009; Lens et al., 1999) and understanding the timing of these major environmental changes is a step closer to understanding the mechanisms by which this occurs.

### 4.4 Developmental instabilities in a changing environment

Our data demonstrate that natural disasters are associated with long‐term developmental disruptions that are most acutely experienced by prenatal individuals. The magnitude of such disruption is evidenced by the elevated levels of FA that persist in individuals more than a decade after the hurricane event they experienced. Thus, the impacts of such disasters are not transient, but instead manifest as lifelong deviations from the normal level of FA observed within the population. Though most individuals that experienced a hurricane prenatally were of a similar gestational age (~8 to 10 weeks gestational age, owing to the relatively consistent annual cycle of both macaque breeding and the tropical hurricane season), individuals who experienced the hurricane both at earlier and later periods of prenatal development exhibit similar levels of FA (Table S1). Such data demonstrate the vulnerability of fetal individuals (and potentially neonatal individuals, though this hypothesis should be explored in future studies with greater numbers of neonates) to developmental instability, and the far‐reaching effects of such disturbances throughout an individual's life.

Hurricane disturbances are complex, dynamic events that can change in both size and intensity while traveling thousands of miles. As hurricane formation is linked—among other external factors—to ocean surface temperatures, both the frequency and magnitude of hurricanes have been tied to global climate change, particularly within tropical oceans between latitudes of 40° S and 40° N (Lugo, 2000; but see Bengtsson et al., 1997), a region referred to as the global hurricane belt. Specifically, global warming has been linked to an increase in the maximum speed of hurricanes, but not the area encapsulated by the hurricane itself (Emanuel, 1997). Similarly, through the use of the Anthropogenic Climate Change Index (ACCI), Holland and Bruyère (2014) demonstrate that the proportion of Category 4 and 5 hurricanes has increased at a rate of ~25% to 30% per °C of global warming—a global signal reproduced in all ocean basins. Thus, as sea surface temperatures continue to rise, it seems reasonable to project an increase in high‐magnitude hurricanes in the coming decades: both within the global hurricane belt and potentially beyond. This phenomenon could have a variety of consequences in that it could (1) expose new populations, previously at low risk of habitat disturbance, to the catastrophic consequences of hurricane events, and (2) subject currently at‐risk populations to the risk of higher magnitude hurricane events. For instance, the macaques of Cayo Santiago recently experienced a third major hurricane event (Hurricane Maria) in 2019, which made landfall as a Category 4 event in September 2017: the most intense strike experienced by the island since 1928 (Zorrilla, 2017). A 63% decrease in vegetation was observed on the island following this hurricane event, resulting in resource scarcity indicated by a peak in adult death rate 1 month after the storm (Testard et al., 2021). Several behavioral and physiological changes were observed as well, especially an increase in the number of social connections (Testard et al., 2021) and an increase in immunological aging in individuals that experienced hurricane Maria (Watowich et al., 2022). This potential danger suggests that the post‐hurricane devastation may be an important stressor for macaques as opposed to solely maternal hormone transfer and underscores the need to better understand the vulnerabilities of populations to natural disasters and better understand the long‐term morphological and fitness implications of catastrophe‐induced environmental stressors.

## 5 CONCLUSION

This study provides evidence that stress from natural disasters during the prenatal period exhibits lasting effects on the primate skeleton, possibly due to increases in maternal stress‐related hormones such as cortisol and glucocorticoids that cause disruptions to typical fetal development. The macaques living on Cayo Santiago are an ideal sample for investigating the effect of hurricane disturbances as most major hurricanes arriving in Puerto Rico have a drastic effect on this island and its inhabitants. Further research in this population is warranted and can provide a clearer picture of the impact of natural disasters on skeletal development, including insights into the effect of social connectedness and nutrition. As climate change continues to create more instability in climatic events, natural disasters are becoming more frequent and severe. These macaques provide a window into the effect such catastrophes can have on both human and nonhuman populations around the world.

## AUTHOR CONTRIBUTIONS

**Ashly N. Romero:** Conceptualization (lead); data curation (lead); formal analysis (supporting); funding acquisition (lead); investigation (lead); methodology (equal); project administration (lead); visualization (lead); writing – original draft (lead); writing – review and editing (equal). **Edwin Dickinson:** Formal analysis (equal); investigation (supporting); methodology (equal); writing – review and editing (equal). **Cassandra M. Turcotte:** Formal analysis (equal); investigation (supporting); methodology (equal); writing – review and editing (equal). **Claire E. Terhune:** Conceptualization (equal); funding acquisition (equal); investigation (equal); resources (lead); supervision (lead); validation (equal); writing – review and editing (equal).

## FUNDING INFORMATION

Funding for this research was provided by P.E.O. International, the University of Arkansas Fulbright College of Arts and Sciences, and the University of Arkansas Department of Anthropology.

## CONFLICT OF INTEREST STATEMENT

All authors declare no competing interests.

### OPEN RESEARCH BADGES

This article has earned Open Data and Open Materials badges. Data and materials are available at https://www.morphosource.org/projects/000373632?locale=en.

## Supporting information


Table S1.

## Data Availability

All 275 cranial 3D surface scans used in this study are on Morphosource.org along with their associated mandibles under the project “Romero Dissertation Scans – CPRC Macaques.” These scans are free for use with the acknowledgement of the Caribbean Primate Research Center that is provided in the project description on Morphosource. The demographic data and Procrustes FA scores associated with these rhesus macaques are available in the Supplementary Information of this publication.
