# Supplementary material for: Skeletal age during hurricane impacts fluctuating asymmetry in Cayo Santiago rhesus macaques
Source: Ecol Evol. 2023 Aug 11;13(8):e10425. doi: 10.1002/ece3.10425 (PMC10421717; doi:10.1002/ece3.10425)
Supplement: Supplementary file 1 — Table S1. [file ECE3-13-e10425-s001.docx]

Table 1. Individuals used in this study that are housed in the Laboratory of Primate Morphology at the University of Puerto Rico Recinto de Ciencias Medicas. Catalog number at LPM, individual id tattoo, exact age at death, and Procrustes FA score are included.

| **Museum Number** | **Tattoo** | **Sex** | **Age at Death** | **Procrustes FA Score** |
| --- | --- | --- | --- | --- |
| CPRCMUS-00008 | EP | M | 6.49 | 0.013367004 |
| CPRCMUS-00011 | FR | F | 0.87 | 0.022349203 |
| CPRCMUS-00019 | 129 | F | 14.17 | 0.010890405 |
| CPRCMUS-00024 | BV | F | 10.28 | 0.014668791 |
| CPRCMUS-00025 | OZ | M | 1.90 | 0.011753388 |
| CPRCMUS-00031 | NL | M | 7.85 | 0.017900907 |
| CPRCMUS-00032 | IW | F | 6.53 | 0.015035026 |
| CPRCMUS-00037 | CD | F | 12.26 | 0.011770711 |
| CPRCMUS-00038 | BP | F | 9.94 | 0.012899127 |
| CPRCMUS-00040 | LV | F | 7.14 | 0.015361101 |
| CPRCMUS-00045 | GP | F | 3.66 | 0.018429749 |
| CPRCMUS-00047 | HP | F | 8.57 | 0.012971885 |
| CPRCMUS-00052 | TK | F | 1.61 | 0.01295964 |
| CPRCMUS-00053 | 011 | F | 12.90 | 0.017790057 |
| CPRCMUS-00054 | YK | F | 2.51 | 0.00996115 |
| CPRCMUS-00059 | T | M | 2.90 | 0.01106533 |
| CPRCMUS-00060 | EJ | M | 6.46 | 0.011976978 |
| CPRCMUS-00061 | KU | M | 4.70 | 0.010712334 |
| CPRCMUS-00062 | HH | F | 4.02 | 0.012473262 |
| CPRCMUS-00065 | R011 | F | 8.20 | 0.014561016 |
| CPRCMUS-00068 | BM | M | 4.87 | 0.017780572 |
| CPRCMUS-00072 | LQ | F | 1.58 | 0.018031538 |
| CPRCMUS-00079 | HA | F | 2.77 | 0.014372127 |
| CPRCMUS-00088 | BT | F | 3.08 | 0.015525268 |
| CPRCMUS-00096 | BX INF 1962 | F | 1.88 | 0.016046454 |
| CPRCMUS-00104 | RB INF 1959 | M | 1.17 | 0.012664266 |
| CPRCMUS-00109 | 078 | F | 11.07 | 0.02159338 |
| CPRCMUS-00112 | BL | F | 7.53 | 0.01463098 |
| CPRCMUS-00114 | R003 | F | 10.21 | 0.012272454 |
| CPRCMUS-00116 | XZ | M | 2.18 | 0.01561476 |
| CPRCMUS-00118 | 9 | M | 3.79 | 0.015386578 |
| CPRCMUS-00120 | Z | F | 5.53 | 0.012507864 |
| CPRCMUS-00127 | 010 | F | 9.15 | 0.01264758 |
| CPRCMUS-00131 | FX | M | 5.49 | 0.009433778 |
| CPRCMUS-00133 | 024 | F | 15.53 | 0.014405662 |
| CPRCMUS-00147 | UG | F | 9.53 | 0.010987812 |
| CPRCMUS-00148 | 393 | M | 1.54 | 0.020520108 |
| CPRCMUS-00153 | OJ | M | 7.62 | 0.011974755 |
| CPRCMUS-00154 | W3 | M | 5.69 | 0.015325055 |
| CPRCMUS-00155 | GQ | M | 7.54 | 0.010915061 |
| CPRCMUS-00160 | 8L | F | 3.94 | 0.014209482 |
| CPRCMUS-00163 | GN | M | 7.93 | 0.015137733 |
| CPRCMUS-00164 | FA | M | 10.51 | 0.014470746 |
| CPRCMUS-00174 | KZ | F | 14.24 | 0.009949127 |
| CPRCMUS-00196 | GK | F | 8.03 | 0.014810395 |
| CPRCMUS-00202 | 106 | F | 17.12 | 0.018504774 |
| CPRCMUS-00219 | XK | F | 9.11 | 0.008834357 |
| CPRCMUS-00220 | H6 | F | 7.16 | 0.012783785 |
| CPRCMUS-00221 | E3 | F | 7.06 | 0.01352445 |
| CPRCMUS-00225 | XP | F | 9.11 | 0.014035421 |
| CPRCMUS-00226 | LG | M | 11.04 | 0.016591855 |
| CPRCMUS-00236 | UI | F | 9.98 | 0.01399345 |
| CPRCMUS-00238 | HJ | F | 13.04 | 0.012768234 |
| CPRCMUS-00244 | DK | M | 13.03 | 0.013026294 |
| CPRCMUS-00245 | OY | M | 7.03 | 0.014333076 |
| CPRCMUS-00247 | XA | M | 10.03 | 0.015686726 |
| CPRCMUS-00258 | Z9 | M | 5.09 | 0.011831418 |
| CPRCMUS-00271 | ZQ | F | 8.04 | 0.014234407 |
| CPRCMUS-00281 | TB | M | 9.91 | 0.016016573 |
| CPRCMUS-00289 | HC | M | 12.05 | 0.015001534 |
| CPRCMUS-00300 | EG | M | 12.21 | 0.014667162 |
| CPRCMUS-00314 | 9U | M | 4.69 | 0.015442058 |
| CPRCMUS-00320 | ZR | M | 8.74 | 0.016918543 |
| CPRCMUS-00321 | 3U | M | 5.70 | 0.012350153 |
| CPRCMUS-00324 | 8N | M | 4.79 | 0.020178916 |
| CPRCMUS-00326 | K | F | 12.53 | 0.014959207 |
| CPRCMUS-00333 | 391 | M | 4.01 | 0.021426327 |
| CPRCMUS-00337 | ZK | M | 9.38 | 0.014239032 |
| CPRCMUS-00338 | 031 | F | 18.25 | 0.011781241 |
| CPRCMUS-00339 | 519 | M | 1.11 | 0.013414805 |
| CPRCMUS-00345 | AH | F | 14.99 | 0.012543485 |
| CPRCMUS-00346 | 286 | F | 3.12 | 0.016198167 |
| CPRCMUS-00349 | 382 | F | 3.43 | 0.018682357 |
| CPRCMUS-00352 | 290 | F | 3.39 | 0.015118887 |
| CPRCMUS-00353 | 253 | F | 4.08 | 0.026100887 |
| CPRCMUS-00354 | 022 | F | 18.64 | 0.014529247 |
| CPRCMUS-00358 | S017 | F | 16.42 | 0.015056478 |
| CPRCMUS-00361 | E2 | M | 8.34 | 0.015948294 |
| CPRCMUS-00364 | DS | M | 14.47 | 0.010501039 |
| CPRCMUS-00366 | 559 | F | 1.21 | 0.01778617 |
| CPRCMUS-00368 | 318 | M | 3.61 | 0.011877415 |
| CPRCMUS-00371 | 201 | M | 5.45 | 0.018517616 |
| CPRCMUS-00374 | XQ | F | 10.39 | 0.012918293 |
| CPRCMUS-00379 | F7 | M | 8.50 | 0.016358766 |
| CPRCMUS-00380 | F8 | M | 8.50 | 0.009634998 |
| CPRCMUS-00381 | FJ | M | 9.63 | 0.014688066 |
| CPRCMUS-00382 | TD | M | 11.58 | 0.013911209 |
| CPRCMUS-00383 | XC | F | 10.44 | 0.011736225 |
| CPRCMUS-00385 | 7J | M | 6.24 | 0.013611381 |
| CPRCMUS-00398 | 9Z | M | 7.91 | 0.02030072 |
| CPRCMUS-00402 | JX | M | 13.90 | 0.014421994 |
| CPRCMUS-00406 | OS | F | 9.53 | 0.015450767 |
| CPRCMUS-00417 | 2C | M | 7.53 | 0.024246441 |
| CPRCMUS-00422 | JI | F | 14.74 | 0.013589156 |
| CPRCMUS-00426 | 489 | M | 3.85 | 0.020593506 |
| CPRCMUS-00427 | 434 | M | 3.11 | 0.017767957 |
| CPRCMUS-00434 | ZB | M | 11.69 | 0.012081041 |
| CPRCMUS-00440 | YB | F | 12.04 | 0.013513306 |
| CPRCMUS-00442 | ZH | M | 11.66 | 0.01675941 |
| CPRCMUS-00447 | 9T | F | 7.96 | 0.009353646 |
| CPRCMUS-00471 | 730 | F | 1.79 | 0.014997439 |
| CPRCMUS-00477 | 737 | F | 0.95 | 0.015211228 |
| CPRCMUS-00478 | 359 | F | 8.51 | 0.00982092 |
| CPRCMUS-00479 | 706 | M | 1.05 | 0.015324666 |
| CPRCMUS-00543 | A05 | M | 0.95 | 0.015003182 |
| CPRCMUS-00562 | 949 | M | 2.81 | 0.01703194 |
| CPRCMUS-00574 | 258 INF 1980 | M | 0.77 | 0.018456009 |
| CPRCMUS-00582 | 495 | M | 9.38 | 0.033535791 |
| CPRCMUS-00593 | B15 | M | 1.75 | 0.012787146 |
| CPRCMUS-00596 | 283 | F | 10.97 | 0.012367505 |
| CPRCMUS-00597 | 306 | F | 11.07 | 0.01774536 |
| CPRCMUS-00616 | C36 | M | 0.93 | 0.009753086 |
| CPRCMUS-00617 | A6 | F | 16.97 | 0.018511927 |
| CPRCMUS-00620 | G8 | F | 16.91 | 0.012914771 |
| CPRCMUS-00637 | 500 | F | 10.64 | 0.010326309 |
| CPRCMUS-00644 | 703 | M | 8.16 | 0.012672132 |
| CPRCMUS-00672 | 258 | F | 14.20 | 0.01400371 |
| CPRCMUS-00684 | V7 | F | 17.94 | 0.013297711 |
| CPRCMUS-00799 | 5D | M | 16.49 | 0.013522309 |
| CPRCMUS-00801 | 648 | M | 8.18 | 0.016835408 |
| CPRCMUS-00806 | 599 | M | 10.47 | 0.010026183 |
| CPRCMUS-00841 | 287 | F | 15.04 | 0.019498109 |
| CPRCMUS-00842 | 606 | F | 10.07 | 0.012555671 |
| CPRCMUS-00848 | 569 | M | 10.50 | 0.011062162 |
| CPRCMUS-00852 | 615 | F | 9.78 | 0.016427653 |
| CPRCMUS-00853 | 643 | F | 8.47 | 0.015168751 |
| CPRCMUS-01213 | A20 | M | 7.64 | 0.01016217 |
| CPRCMUS-01216 | 4T | M | 18.73 | 0.014735844 |
| CPRCMUS-01231 | 619 | M | 12.10 | 0.013710497 |
| CPRCMUS-01232 | 604 | F | 11.84 | 0.014766653 |
| CPRCMUS-01233 | 438 | F | 12.35 | 0.012648259 |
| CPRCMUS-01243 | B67 | M | 7.23 | 0.015703467 |
| CPRCMUS-01246 | B23 | M | 7.55 | 0.020389381 |
| CPRCMUS-01252 | 962 | M | 9.46 | 0.01455131 |
| CPRCMUS-01570 | 9L | M | 19.64 | 0.012745899 |
| CPRCMUS-01571 | B06 | M | 7.53 | 0.012608427 |
| CPRCMUS-01573 | B61 | M | 7.37 | 0.013326985 |
| CPRCMUS-01574 | 894 | M | 9.52 | 0.016318173 |
| CPRCMUS-01575 | 564 | M | 14.64 | 0.024512947 |
| CPRCMUS-01579 | 996 | M | 7.94 | 0.012289452 |
| CPRCMUS-01580 | 935 | M | 9.70 | 0.014812385 |
| CPRCMUS-01587 | B65 | F | 7.63 | 0.011896158 |
| CPRCMUS-02029 | B74 | F | 7.90 | 0.017009446 |
| CPRCMUS-02031 | 676 | F | 12.51 | 0.013762115 |
| CPRCMUS-02032 | 795 | M | 11.50 | 0.018752461 |
| CPRCMUS-02039 | 818 | M | 10.90 | 0.011256605 |
| CPRCMUS-02092 | D13 | M | 8.11 | 0.014391824 |
| CPRCMUS-02961 | 436 | M | 18.39 | 0.020164868 |
| CPRCMUS-02965 | 504 | M | 18.66 | 0.012780984 |
| CPRCMUS-02968 | 405 | F | 21.92 | 0.019924961 |
| CPRCMUS-02971 | WK | M | 29.10 | 0.017420707 |
| CPRCMUS-03007 | 348 | F | 20.10 | 0.011900383 |
| CPRCMUS-03015 | 568 | M | 18.38 | 0.013703612 |
| CPRCMUS-03017 | 976 | F | 10.59 | 0.012171821 |
| CPRCMUS-03023 | 941 | F | 12.97 | 0.010821029 |
| CPRCMUS-03024 | 787 | M | 14.37 | 0.016560131 |
| CPRCMUS-03028 | C28 | M | 8.89 | 0.017256252 |
| CPRCMUS-03029 | 974 | F | 12.02 | 0.010547408 |
| CPRCMUS-03034 | D53 | M | 8.65 | 0.018993334 |
| CPRCMUS-03044 | D84 | M | 8.52 | 0.011854031 |
| CPRCMUS-03060 | 798 | F | 14.42 | 0.011164568 |
| CPRCMUS-03129 | H50 | M | 7.71 | 0.016442575 |
| CPRCMUS-03196 | G24 | F | 7.03 | 0.019998767 |
| CPRCMUS-03214 | K12 | M | 7.27 | 0.01548637 |
| CPRCMUS-03260 | I07 | F | 8.73 | 0.014684494 |
| CPRCMUS-03287 | D68 | M | 13.32 | 0.017659374 |
| CPRCMUS-03291 | 971 | F | 15.61 | 0.013659002 |
| CPRCMUS-03307 | FB | F | 31.42 | 0.011517633 |
| CPRCMUS-03308 | F19 | F | 13.19 | 0.010364663 |
| CPRCMUS-03317 | O78 | F | 7.62 | 0.012055998 |
| CPRCMUS-03319 | E67 | F | 11.25 | 0.022217672 |
| CPRCMUS-03341 | K82 | M | 7.82 | 0.01216616 |
| CPRCMUS-03348 | B03 | F | 13.01 | 0.012027313 |
| CPRCMUS-03450 | H42 | M | 11.50 | 0.011753782 |
| CPRCMUS-03453 | S11 | F | 7.44 | 0.019820857 |
| CPRCMUS-03477 | S09 | M | 4.86 | 0.013255979 |
| CPRCMUS-03531 | 725 | F | 17.41 | 0.013278241 |
| CPRCMUS-03533 | J43 | M | 10.24 | 0.015784153 |
| CPRCMUS-03600 | J61 | F | 9.58 | 0.011363093 |
| CPRCMUS-03602 | 439 | M | 23.89 | 0.01106301 |
| CPRCMUS-03603 | I95 | F | 9.71 | 0.018698302 |
| CPRCMUS-03630 | L93 | F | 7.09 | 0.009868347 |
| CPRCMUS-03637 | V61 | F | 6.36 | 0.017355576 |
| CPRCMUS-03646 | D10 | M | 15.16 | 0.0188552 |
| CPRCMUS-03689 | V97 | F | 0.98 | 0.01248337 |
| CPRCMUS-03694 | R52 | M | 2.97 | 0.013505994 |
| CPRCMUS-03697 | X62 | F | 2.22 | 0.042164742 |
| CPRCMUS-03745 | 87B | F | 1.11 | 0.012910114 |
| CPRCMUS-03766 | Z09 | M | 1.66 | 0.011536106 |
| CPRCMUS-03787 | 26B | F | 2.09 | 0.014166141 |
| CPRCMUS-03811 | T93 | F | 2.84 | 0.012052053 |
| CPRCMUS-03853 | 10K | M | 2.28 | 0.013440044 |
| CPRCMUS-03906 | H66 | M | 10.72 | 0.015090152 |
| CPRCMUS-03909 | J88 | M | 17.34 | 0.013789214 |
| CPRCMUS-03910 | 86A | M | 7.69 | 0.015921142 |
| CPRCMUS-03911 | H47 | M | 12.33 | 0.012291091 |
| CPRCMUS-03913 | 845 | F | 23.47 | 0.013049958 |
| CPRCMUS-03917 | E04 | F | 18.69 | 0.034677137 |
| CPRCMUS-03919 | H57 | F | 8.28 | 0.012471204 |
| CPRCMUS-03921 | 08I | F | 5.08 | 0.011924043 |
| CPRCMUS-03929 | O50 | F | 4.06 | 0.013647602 |
| CPRCMUS-03930 | L43 | M | 14.35 | 0.012892915 |
| CPRCMUS-03931 | J95 | F | 17.41 | 0.012673017 |
| CPRCMUS-04078 | 84O | M | 1.27 | 0.015381109 |
| CPRCMUS-04112 | 08A | M | 11.31 | 0.015739067 |
| CPRCMUS-04147 | S37 | F | 14.87 | 0.014534706 |
| CPRCMUS-04164 | 59B | M | 9.83 | 0.013789383 |
| CPRCMUS-04168 | 39C | M | 10.07 | 0.013594749 |
| CPRCMUS-04176 | H71 | F | 19.86 | 0.01491666 |
| CPRCMUS-04181 | 679 | F | 28.93 | 0.011044323 |
| CPRCMUS-04187 | X67 | F | 13.55 | 0.012380091 |
| CPRCMUS-04248 | 56O | F | 3.59 | 0.016229665 |
| CPRCMUS-04276 | 21I | M | 5.99 | 0.019755542 |
| CPRCMUS-04300 | 84F | M | 9.74 | 0.014640524 |
| CPRCMUS-04306 | 33A | M | 10.52 | 0.016200135 |
| CPRCMUS-04310 | H45 | F | 20.94 | 0.012884548 |
| CPRCMUS-04314 | 23H | M | 7.63 | 0.011955276 |
| CPRCMUS-04316 | B60 | F | 23.39 | 0.012861147 |
| CPRCMUS-04318 | O53 | F | 17.51 | 0.024407693 |
| CPRCMUS-04328 | J99 | F | 18.81 | 0.008418087 |
| CPRCMUS-04332 | 03G | M | 6.60 | 0.011780228 |
| CPRCMUS-04335 | 85H | M | 5.84 | 0.016878771 |
| CPRCMUS-04341 | 47K | F | 5.53 | 0.017876095 |
| CPRCMUS-04343 | 28E | M | 8.74 | 0.021264279 |
| CPRCMUS-04409 | 81L | F | 3.97 | 0.015942977 |
| CPRCMUS-04423 | 08N | M | 6.82 | 0.01510583 |
| CPRCMUS-04426 | 31A | F | 14.81 | 0.014949547 |
| CPRCMUS-04431 | 25O | F | 6.86 | 0.018428265 |
| CPRCMUS-04432 | 48B | F | 13.70 | 0.013401117 |
| CPRCMUS-04433 | K81 | F | 18.81 | 0.015424828 |
| CPRCMUS-04439 | T98 | F | 15.88 | 0.02108866 |
| CPRCMUS-04458 | 74E | F | 7.15 | 0.018592975 |
| CPRCMUS-04459 | J79 | F | 17.27 | 0.036047244 |
| CPRCMUS-04461 | H74 | F | 21.91 | 0.016476764 |
| CPRCMUS-04469 | 95A | F | 13.95 | 0.014800614 |
| CPRCMUS-04470 | G68 | F | 20.15 | 0.011711748 |
| CPRCMUS-04480 | X87 | F | 15.90 | 0.013843321 |
| CPRCMUS-04485 | 97S | M | 3.26 | 0.013204142 |
| CPRCMUS-04486 | R75 | F | 17.98 | 0.015446746 |
| CPRCMUS-04487 | Z99 | F | 14.75 | 0.014804412 |
| CPRCMUS-04493 | 39S | F | 4.19 | 0.01002932 |
| CPRCMUS-04497 | 79O | M | 5.25 | 0.01391728 |
| CPRCMUS-04507 | H79 | M | 16.38 | 0.013209255 |
| CPRCMUS-04508 | 27C | F | 9.30 | 0.011354414 |
| CPRCMUS-04509 | 25I | M | 10.66 | 0.011844786 |
| CPRCMUS-04514 | 90S | F | 5.83 | 0.012082242 |
| CPRCMUS-04532 | 6C8 | F | 2.95 | 0.014787796 |
| CPRCMUS-04538 | 23V | F | 4.03 | 0.023993184 |
| CPRCMUS-04540 | 94V | F | 4.84 | 0.011720183 |
| CPRCMUS-04543 | X53 | F | 3.46 | 0.019084778 |
| CPRCMUS-04548 | 99I | F | 11.29 | 0.012028326 |
| CPRCMUS-04571 | 80J | F | 1.38 | 0.024895019 |
| CPRCMUS-04622 | O55 | F | 19.68 | 0.019675947 |
| CPRCMUS-04631 | 01P | F | 7.90 | 0.011312285 |
| CPRCMUS-04634 | 35J | F | 10.72 | 0.012987542 |
| CPRCMUS-04640 | 37T | F | 4.86 | 0.013660908 |
| CPRCMUS-04652 | 43K | F | 4.24 | 0.017970222 |
| CPRCMUS-04654 | 985 | F | 24.11 | 0.014469128 |
| CPRCMUS-04656 | F25 | F | 24.06 | 0.014878948 |
| CPRCMUS-04669 | 874 | F | 19.74 | 0.012220138 |
| CPRCMUS-04675 | V24 | F | 11.05 | 0.01259001 |
| CPRCMUS-04778 | 89C | F | 13.63 | 0.015737357 |
| CPRCMUS-04780 | V44 | F | 17.57 | 0.010885244 |
| CPRCMUS-04781 | 91K | F | 8.70 | 0.015105153 |
| CPRCMUS-04782 | 29I | F | 10.00 | 0.013420656 |
| CPRCMUS-04784 | 04O | F | 9.23 | 0.022943777 |
| CPRCMUS-04786 | K86 | M | 7.39 | 0.0142275 |
| CPRCMUS-04788 | 33C | F | 15.14 | 0.012950718 |
| CPRCMUS-04795 | 47L | F | 8.89 | 0.015967086 |
| CPRCMUS-04810 | 35O | F | 11.33 | 0.011238466 |
| CPRCMUS-04811 | 26P | M | 11.37 | 0.017471067 |
| CPRCMUS-04812 | 28N | F | 12.44 | 0.012690708 |
| CPRCMUS-04813 | 9A9 | M | 7.64 | 0.018685941 |
| CPRCMUS-04815 | 17T | M | 9.42 | 0.017498986 |
| CPRCMUS-04817 | 45F | F | 16.12 | 0.015399439 |
